# Recreational vessels without Automatic Identification System (AIS) dominate anthropogenic noise contributions to a shallow water soundscape

**DOI:** 10.1038/s41598-019-51222-9

**Published:** 2019-10-29

**Authors:** Line Hermannsen, Lonnie Mikkelsen, Jakob Tougaard, Kristian Beedholm, Mark Johnson, Peter T. Madsen

**Affiliations:** 1 Zoophysiology, Department of Bioscience, Aarhus University, Aarhus, Denmark; 2 Marine Mammal Research, Department of Bioscience, Aarhus University, Aarhus, Denmark; 3 Sea Mammal Research Unit, University of St. Andrews, St. Andrews, United Kingdom; 4 Aarhus Institute of Advanced Studies, Aarhus University, Aarhus, Denmark

**Keywords:** Animal behaviour, Environmental impact, Marine biology

## Abstract

Recreational boating is an increasing activity in coastal areas and its spatiotemporal overlap with key habitats of marine species pose a risk for negative noise impacts. Yet, recreational vessels are currently unaccounted for in vessel noise models using Automatic Identification System (AIS) data. Here we conduct a case study investigating noise contributions from vessels with and without AIS (non-AIS) in a shallow coastal area within the Inner Danish waters. By tracking vessels with theodolite and AIS, while recording ambient noise levels, we find that non-AIS vessels have a higher occurrence (83%) than AIS vessels, and that motorised recreational vessels can elevate third-octave band noise centred at 0.125, 2 and 16 kHz by 47–51 dB. Accordingly, these vessels dominated the soundscape in the study site due to their high numbers, high speeds and proximity to the coast. Furthermore, recreational vessels caused 49–85% of noise events potentially eliciting behavioural responses in harbour porpoises (AIS vessels caused 5–24%). We therefore conclude that AIS data would poorly predict vessel noise pollution and its impacts in this and other similar marine environments. We suggest to improve vessel noise models and impact assessments by requiring that faster and more powerful recreational vessels carry AIS-transmitters.

## Introduction

Motorised vessels are the most widespread source of anthropogenic underwater noise and may be causing behavioural responses and acoustic masking in a wide range of marine species worldwide^1–3^. Globally, the United Nations convention UNCLOS^4^ requires members to “prevent, reduce and control pollution at sea” including energy which covers underwater noise. In Europe, efforts to monitor and manage vessel noise pollution are further embodied in the Marine Strategy Framework Directive^5^ (MSFD, descriptor 11.2), which mandates member states to ensure that underwater noise levels do not exceed thresholds that compromise good environmental status^6^. To meet legislative requirements and manage underwater noise pollution, cost-effective strategies to monitor vessel noise and its potential effects on marine life are being developed. An increasingly popular tool to estimate vessel noise loads is predictive modelling based on ship movement data from the Automatic Identification System (AIS)^7–9^. Vessels with AIS periodically broadcast a unique identifier along with their position, course and speed, allowing for model predictions of noise loads around the vessel as it moves^8^. However, globally AIS-transmitters are only required on larger ships (>300 gross tonnage), passenger vessels and large fishing vessels^10,11^. Accordingly, the more than 30 million recreational vessels worldwide^12^ are not required to have an AIS, and these non-AIS vessels are therefore currently not accounted for in AIS-based models^13,14^. This is despite that recreational vessels may emit considerable levels of broadband noise^15,16^ with potential negative effects on marine species with a wide range of hearing capabilities^17^, including fish^18,19^, crustaceans^20^, sea turtles^21^ and cetaceans^14,16,22^. Cavitation noise from motorboats can extend to mid-to-high frequencies up to and beyond 100 kHz^16^, and therefore also overlap with toothed whale hearing and echolocation^1,23^. Because of this, noise generated by motorboats has been linked to avoidance, changes in dive pattern, altered vocal behaviour and decreased foraging in small toothed whales^24–27^. To manage vessel noise pollution and minimise negative impacts on marine species, it is therefore important to assess whether predictive models based only on AIS data provide reliable estimates of actual vessel noise, or whether these models risk underestimating noise impacts on protected wildlife in some marine environments.

Shallow, coastal areas are key habitats for many marine species, but are likely particularly prone to underestimations of vessel noise levels by AIS models, as recreational vessels are common close to the coast, whereas commercial vessels with AIS typically pass in offshore shipping lanes^28^. In Europe, 46% of coastal waters (within 5 km of the coast) have water depths shallower than 20 m^29,30^. Shallow water depths act as high pass filters^31^ that dramatically increase the transmission loss of low-frequency sound, including low-frequency components in vessel noise. Depending on the seabed composition, the cut-off frequency in water depths of 20 m will be between 19 and 135 Hz^23^. As a consequence, low-frequency noise from large distant vessels may be less of a concern in shallow waters, whereas mid-to-high frequency noise from closer sources, such as small recreational vessels without AIS, may contribute significant to local soundscapes with potential negative effects on marine species that depend on these habitats to forage, breed and/or rest^18,20,21,32^.

With more than 4,500 marinas and 6 million recreational vessels in Europe^12^, as well as a high shipping density^28^, coastal areas with mixed vessel traffic are common, and vessel density is especially high during summer when recreational boating peaks^28^. Here we conduct a case study, recording underwater noise levels in a shallow coastal area with mixed vessel traffic, while monitoring all nearby vessels, to assess how well vessel noise levels can be explained by the presence of AIS and non-AIS vessels. Specifically we sought to address the question of whether noise predictions based solely on AIS data can cause significant underestimations of vessel noise pollution and its impacts on coastal marine environments. Since the small toothed whale, the harbour porpoise (*phocoena phocoena*) is well known to be sensitive to anthropogenic noise pollution^33–35^ and at the same time is common in many coastal areas in the northern hemisphere^36,37^, including our study area^34^, we used this protected marine species^38,39^ as a model species for assessing noise impacts. Response thresholds of porpoises to noise from three published studies were used to evaluate the implications for impact assessment of using either AIS or non-AIS vessel data or both. We show that non-AIS vessels dominated the vessel occurrence in this area, and that noise in third-octave bands centred at 0.125, 2 and 16 kHz was mainly caused by motorised recreational vessels without AIS. Given the high density, high speeds and broadband noise emissions of motorised non-AIS vessels, we find that recreational vessels were also the main contributors of noise exceeding response thresholds of porpoises.

## Materials and Methods

### Study area

The study was conducted on August 13–22, 2016, in a shallow coastal area (mostly <20 m deep), in the Inner Danish Waters east of the peninsula Helgenaes, Denmark (56°5.9N, 10°32.4E; Supplementary Fig. S1). The field site is close to a busy shipping lane (Supplementary Fig. S2) and several recreational marinas, and consequently has mixed vessel traffic. This study area is therefore likely broadly representative of many densely populated shallow coastal areas worldwide. Harbour porpoises are known to be present in this area during summer months^34^.

### Underwater noise recordings

Recordings of underwater noise were made with a stationary acoustic recorder (SoundTrap, model ST300HF, Ocean Instruments, Auckland, New Zealand) sampling at a rate of 576 kHz (clipping level 172 dB re 1 µPa). The recorder was deployed 670 ± 5 m from the coast, and was moored at a depth of 7.5 ± 1 m, 1.5 m above the seafloor (i.e. water depth was 9 ± 1 m., further details in the Supplementary Material). A sound speed profile at the deployment site indicated a well-mixed water column (1497–1499 m/s; SVP-14 unit, Reson A/S, Slangerup, Denmark).

Sound recordings were corrected for clock drift and quantified as 1 s average third-octave levels (TOLs) in the bands from 0.063 to 125 kHz (custom script, MATLAB version 2016a). Third-octave bands centred at 0.125 kHz, 2 kHz and 16 kHz were chosen for further analysis based on the following motivation: 0.125 kHz is one of the two frequency bands defined for vessel noise monitoring by the MSFD^40^; the 2 kHz band has been suggested as a better proxy for quantifying mid-to-high frequency components in vessel noise in shallow waters by the international BIAS group^9^; and the 16 kHz band has recently been used in quantifying behavioural responses of harbour porpoises to vessel noise^35^. The MSFD specifies third octave measurement bands at 0.063 kHz and 0.125 kHz, but only the latter was evaluated here, since the shallow water depth will cause a much poorer propagation of noise at 0.063 kHz (e.g. the cut-off frequency for a 20 m deep, soft bottom habitat is ~0.135 kHz^23^) and the 0.125 kHz band was therefore chosen as the best representative of the two MSFD bands. Self-noise of the recorder was measured to be 66, 60 and 66 dB re 1 µPa (root mean squared, RMS) in the three frequency bands, respectively. Ambient noise levels were estimated by the 5^th^ percentile (i.e. 95% exceedance level) for each third-octave band throughout the study period.

### Vessel positions

AIS data were obtained from the Danish Maritime Authority and processed to contain only vessels that passed within compass bearings 31–263° of the sound recorder, to exclude vessel positions shaded by land. Theodolite tracking of vessels was conducted from a high point (47 m above sea level, same position as in^34^) in periods with no rain and WMO sea state <4. Tracking of the closest motorised recreational vessel was prioritised (see Supplementary Material for further details about theodolite tracking methods). Only vessels within 2 km of the recorder were accepted for further analysis for two reasons; (1) the contribution of noise from vessels beyond 2 km was expected to be small due to the high transmission loss over this range; (2) the localization accuracy of the theodolite falls off steeply beyond 2 km given the observation height. Smaller vessels were blocked visually by land to the east of the observation point and so could not be tracked beyond about 500–1000 m of the recorder in that direction. However, noise contributions from these vessels were also shaded by a sandbank meaning that errors in assessing noise levels caused by omitting these vessels were likely small.

### Vessel tracks

AIS and theodolite tracking points were interpolated to 1 s intervals using a Kalman filter (Supplementary Fig. S3). Interpolated data helped to assess which vessel was closest to the recorder at any time, and to determine when a vessel was within the 2 km range criterion (Supplementary Fig. S1). The theodolite tracks of small vessels were compared to AIS tracks to check whether they had an AIS (Supplementary Fig. S4), in which case they were assigned to the AIS-vessel group. Based on this, the error in theodolite range was estimated to be a maximum of 200 m within the study area (Supplementary Fig. S4), assuming that AIS data points reflected true vessel positions. Tracking periods were divided into five categories based on vessel tracks: (1) periods where only AIS vessels were present; (2) periods with one or more vessels with AIS together with one or more non-AIS vessels; (3) periods with at least one motorised non-AIS vessel (not including sailboats potentially powered by motor), but no vessels carrying AIS within 2 km; (4) periods with only sailboats without AIS present; and (5) periods with no vessels within 2 km of the recorder (see also Supplementary Table S1 and Fig. S5). Periods with presence of at least one motorised vessel, either AIS, non-AIS or both (categories 1–3), were investigated further to assess how received noise levels correlated with range to the closest vessel. Periods in which another motorised vessel was within 200 m of the closest vessel were excluded to increase the likelihood of the closest vessel being the main contributor to the recorded noise level (see Supplementary Material p. 4–5). This criterion was based on the estimated maximum theodolite error within our study area of 200 m (Supplementary Fig. S4). Sailboats were assumed to contribute insignificantly to the recorded noise levels, when not being the closest vessel, due to their low speeds during motor propulsion. Because of this, we chose not to account for them in the 200 m criterion, and they could therefore have been within 200 m of the closest motorised vessel.

### Statistical tests

To test whether vessel type (AIS or non-AIS) had an explanatory effect on recorded TOLs, we ran a generalised linear mixed-effect model (GLMM) for each of the three frequency bands (0.125, 2 and 16 kHz), which accounted for the effects of range, vessel type and speed (fixed effects). The recording day was added as a random effect. TOLs for each frequency were normally distributed, which was added as input to the function (*fitglme* in MATLAB version R2016a). Range was log-transformed as the relationship between range and TOLs was non-linear (Fig. 5), and to obtain normally distributed residuals.

**Figure 1:**
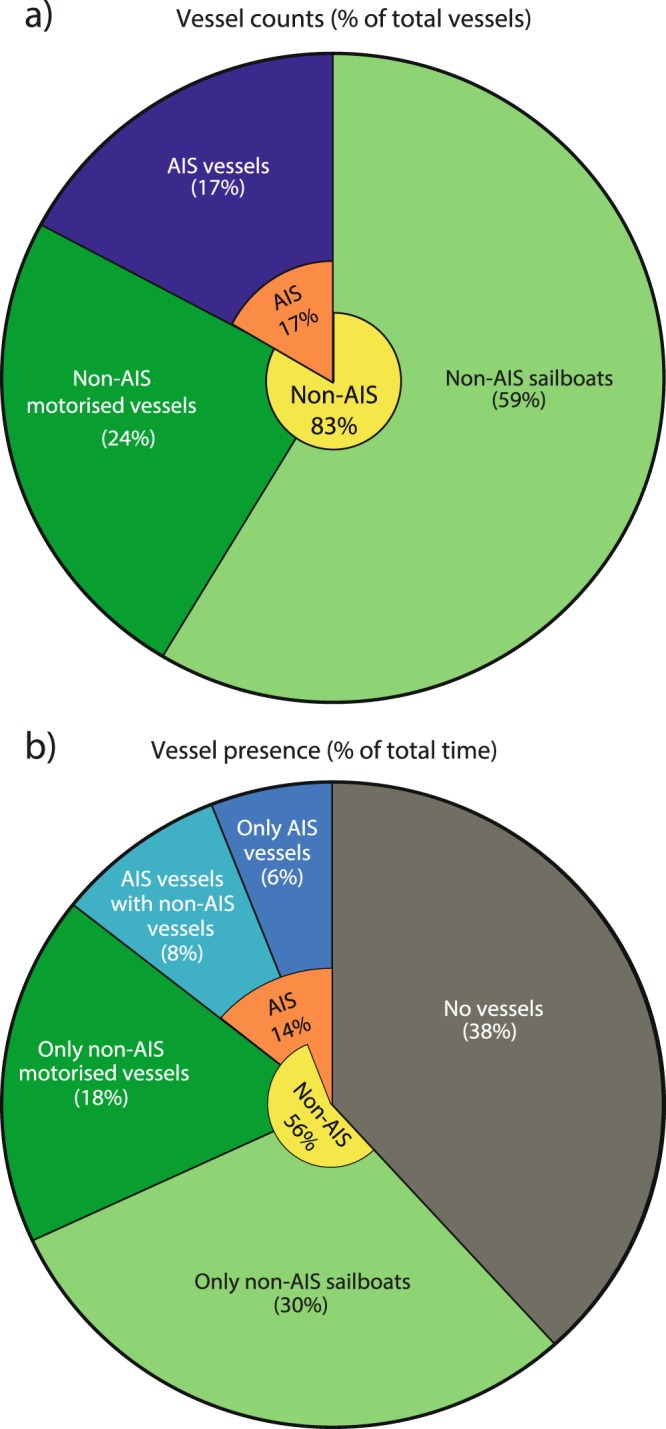
The time distribution of vessel presence and passes. (**a**) The percentage of vessel passes of different vessel types (198 total passes) and (**b**) the time fraction that vessels of each type were present within the study area.

**Figure 2:**
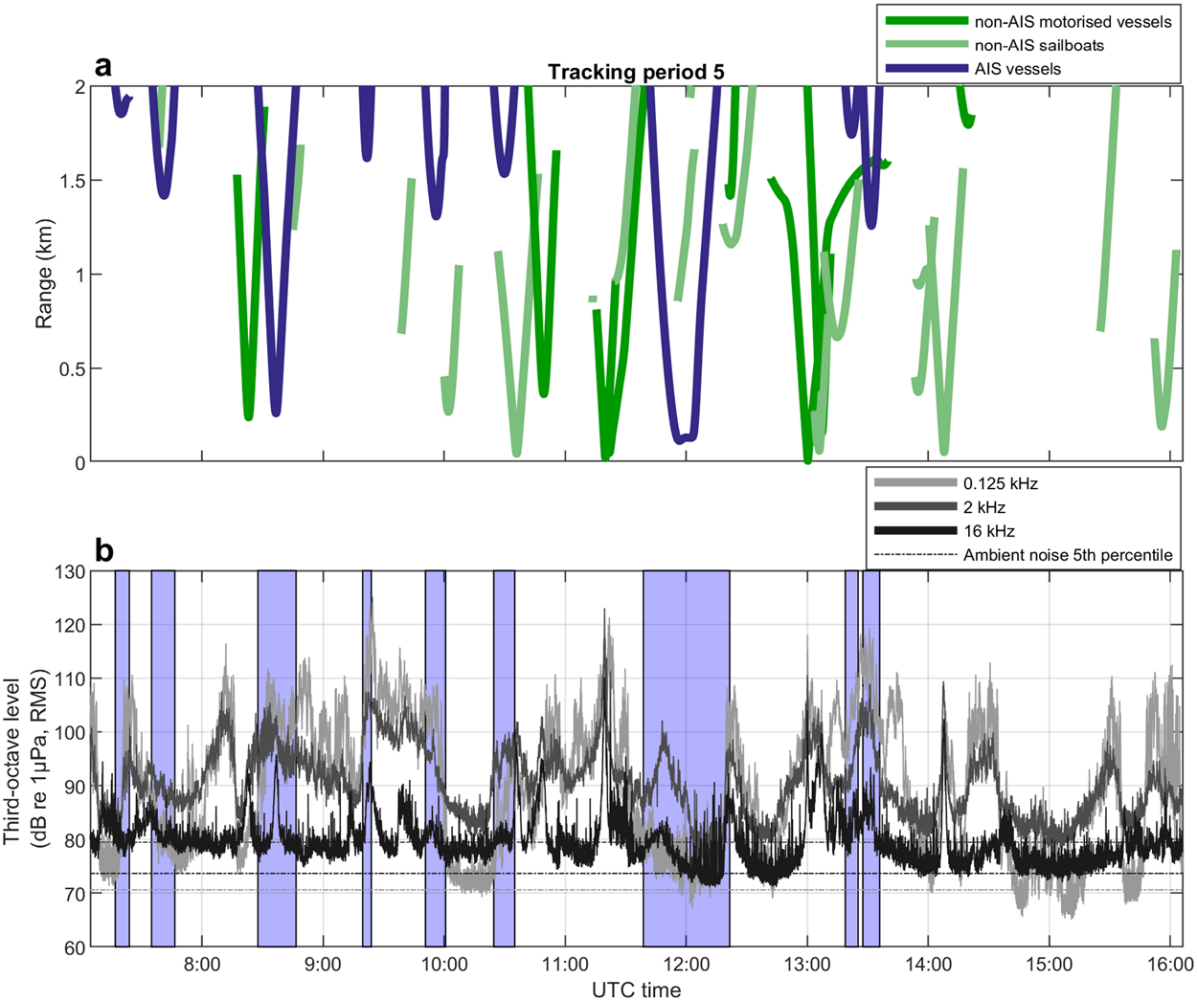
Vessel tracks and underwater noise levels. (**a**) Vessel tracks of AIS (blue) and non-AIS vessels (green) within the study area during one tracking period together with (**b**) recorded underwater noise levels at the three selected frequency bands 0.125, 2 and 16 kHz over the same time. Ambient noise levels calculated as 5^th^ percentiles over the entire study period are plotted as dashed coloured lines in (**b**) with values shown on the right side of the plot. Time periods on (**b**), marked with blue shaded areas, indicate times at which an AIS vessel was present according to vessel tracks shown in (**a**).

**Figure 3:**
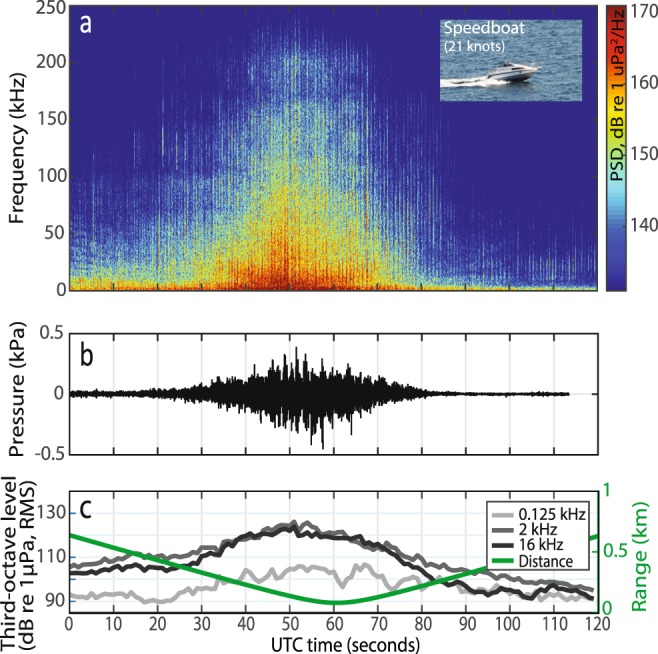
Passage of a speedboat (without AIS) at high speed (mean speed of 21 knots). (**a**) A spectrogram composed of power spectral densities (PSD, i.e. power per 1 Hz band; colour bar on right side), (**b**) waveform, (**c**) Third-octave levels (TOLs) centred at 0.125, 2 and 16 kHz and the range to the recorder (green). A photo of the recorded vessel is shown in the top right corner on (**a**).

**Figure 4:**
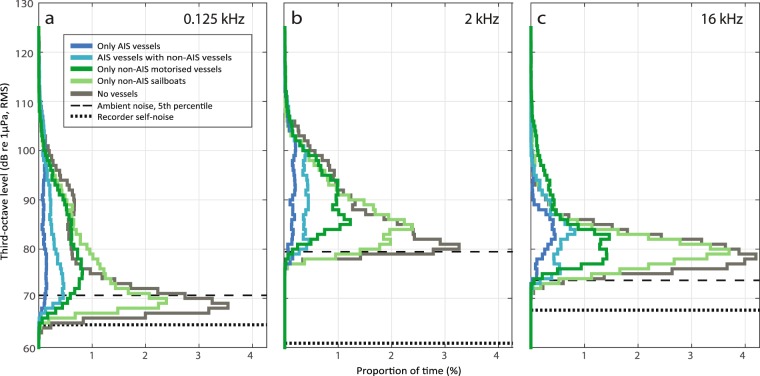
Histograms of recorded noise levels during presence of different vessel types. Subplots show recorded noise in third-octave bands centred at (**a**) 0.125 kHz, (**b**) 2 kHz and (**c**) 16 kHz. Colours represent times with only AIS vessels present (dark blue), both AIS and non-AIS vessels present (light blue), only non-AIS vessels present with at least one being motorised (dark green), only non-AIS sailboats present (light green) or no vessels present (grey). The x-axis shows the proportion of the time (%) that each third-octave band level (1 dB intervals from 60 to 130 dB re 1µPa, RMS) occurs within each group. Ambient noise (5^th^ percentile) is plotted as dashed horizontal lines.

**Figure 5:**
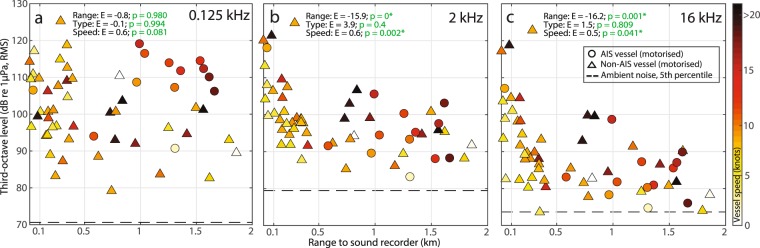
Recorded noise levels as a function of the range to the closest vessel. Subplots show noise in third-octave bands centred at (**a**) 0.125 kHz, (**b**) 2 kHz and (**c**) 16 kHz. Marker shapes show whether the closest vessel was a motorised AIS vessel (circle) or a motorised non-AIS vessel (triangle), while marker colours indicate the speed of the vessel (colour bar on the right). Ambient noise levels (5^th^ percentiles) are also plotted (dashed horizontal lines). Results of the generalised linear mixed-effects models (GLMMs) are shown as estimates (E) and p-values for range, vessel type and speed in each subplot for the three frequency bands (a-c). N = 58 vessel observations.

### Potential impacts on harbour porpoises

To evaluate the incidence of potentially disturbing levels of broadband vessel noise in a shallow coastal area, we use the small toothed whale, the harbour porpoise (*Phocoena phocoena*), as a model species. Harbour porpoises require strict protection and maintenance of their breeding and resting areas^38,39^ and they inhabit shallow, coastal water habitats^36,37^. A number of studies have investigated responses of porpoises to vessel noise and found significant behavioural alterations^35,41,42^. To estimate the potential effects of vessel noise, we applied response thresholds from three published studies to our recordings to identify noise events that could be expected to elicit behavioural responses in porpoises (see Supplementary Material for further details and a description of how these thresholds were applied to the data). The first threshold was from Dyndo *et al*.^41^ which documented porpoising behaviour induced by M-weighted broadband (25 Hz–80 kHz) vessel noise at levels >123 dB re 1 µPa (RMS, 30 s window). The second threshold was calculated based on Tougaard *et al*.^43^, who compiled data from multiple studies of porpoise responses to impulsive sounds and reported a generalised threshold for negative phonotaxis in porpoises at 45 dB above their hearing threshold. We assume that this response threshold (+45 dB sensation level) also applies to broadband, continuous noise, including vessel noise. The third threshold was from Wisniewska *et al*.^35^, who showed a significant reduction in foraging of wild porpoises, when noise exceeded 96 dB re 1 µPa (RMS) in a third-octave band at 16 kHz. For each above-threshold event, the corresponding vessel types in the area were determined from the tracks recorded at the same time (see example in Supplementary Fig. S6).

## Results

### Vessel presence

A total of 198 vessels passed within 2 km of the sound recorder during the observation intervals (Supplementary Table S1), that summed up to a total observation time of 45.6 hours (Supplementary Table S2). Of these, 34 vessels (17%) provided AIS information, while 164 vessels (83%) did not have AIS and were therefore tracked visually with theodolite (Fig. 1a). Non-AIS vessels comprised 46 recreational vessels (e.g. motorboats and speedboats), one fishing vessel, one navy vessel and 116 sailboats (Supplementary Table S2). Of the 116 sailboats, 40 had no sails rigged and so were under motor power and therefore generating noise. The remaining 76 sailboats had one or multiple sails rigged, but it could not be ruled out that they also had their engines on. Using interpolated vessel tracks to estimate time spent for each vessel in the study area, non-AIS vessels were present during 56% of the tracking period (Fig. 1b). In comparison, AIS vessels were present 14% of the time. In 8% of the tracking period, AIS vessels were present together with non-AIS vessels, and both may have contributed to the recorded noise. A few of the instances recorded as having no vessels could have contained noise contributions from non-AIS sailboats, since they did not all have full tracks out to 2 km (Fig. 2a). In comparison, all AIS vessels had complete tracks, as AIS data were always available beyond the study area, and their presence were therefore correctly assessed.

### Recorded noise levels

Noise was quantified in third-octave bands centred at 0.125, 2 and 16 kHz and correlated with vessel presence in the study area (Fig. 2). This shows that peaks in noise correlated well with passing vessels, but also highlights that high noise levels were often recorded when only non-AIS vessels were present in the area (times outside blue boxes in Fig. 2b), Generally during vessel presence, noise in the 2 kHz third-octave band exceeded noise levels at 0.125 and 16 kHz (Supplementary Fig. S7). In contrast, peaks in the 0.125 kHz TOLs did not consistently correlate with vessel presence (Figs 2, S7), and had weaker correlations with high frequency noise at 16 kHz (Pearson’s correlation coefficient = 0.32) than did peaks in the 2 kHz band (Pearson’s correlation coefficient = 0.65, Supplementary Fig. S8). A two-minute recording of a speedboat at close range (80–630 m; Fig. 3) demonstrates that small recreational vessels, which seldom carry an AIS, can generate high levels of broadband noise, and confirms the pattern of noise levels in the 2 kHz third-octave band exceeding 0.125 kHz noise. The underwater noise emissions from this speedboat extended to at least 150 kHz (Fig. 3a) and led to an increase in ambient noise levels of 47 and 50 dB in third-octave bands centred at 2 kHz (max level 126 dB re 1 µPa RMS, ambient noise level 79 dB re 1 µPa RMS) and 16 kHz (max level 124 dB re 1 µPa RMS, ambient noise level 74 dB re 1 µPa). The noise level at 0.125 kHz was elevated by 36 dB (max level 107 dB re 1 µPa RMS, ambient noise level 71 dB re 1 µPa RMS), and noise levels in this band were consistently lower than in the 2 kHz third-octave band and mostly below 16 kHz TOLs (Fig. 3c). Overall, the high noise emissions combined with the high presence of motorised non-AIS vessels in this area (Fig. 1, Supplementary Table S2) resulted in recreational vessels being the dominant contributors of noise across the three selected third-octave bands, giving rise to elevations in ambient noise of up to 55, 47 and 51 dB in the 0.125, 2 and 16 kHz bands, respectively (Fig. 4). However, the GLMM results (Fig. 5, full model results in Supplementary Table S3) show that vessel type did not significantly explain the recorded TOLs for any of the three frequencies. Instead TOLs at 2 and 16 kHz were found to be significantly correlated with vessel range and vessel speeds, with the highest TOLs found at close range and high speeds. TOLs at 0.125 kHz showed no significant correlation with vessel range and speed, and none of the interaction terms were found to be significant.

### Potential impacts on harbour porpoises

**Figure 6:**
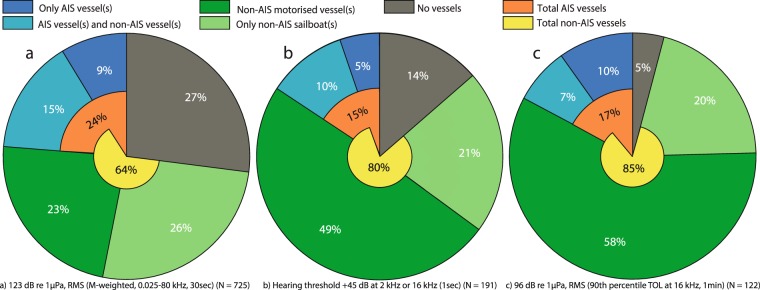
Presence of different vessel groups (colours) at the time of noise events expected to elicit behavioural responses in harbour porpoises. Response thresholds are based on results from three published studies; (**a**) Dyndo *et al*.^41^, (**b**) Tougaard *et al*.^43^ and (**c**) Wisniewska *et al*.^35^. For each threshold the total percentage of events correlating with presence of AIS vessels (orange) and non-AIS vessels (yellow) are also shown, with an overlap due to cases where both AIS and non-AIS vessels are present (light blue). The number of high noise events (N) exceeding each specific threshold is shown at the bottom.

Based on three previous studies, a porpoise at the location of our recorder was estimated to would have experienced 122, 191 or 725 events with noise levels high enough to elicit a behavioural response (Fig. 6). Depending on the response threshold of the animal, this corresponds to behavioural responses 3, 4 or 16 times/hour. Vessels within our study area were estimated to account for 63–95% of these above-threshold events, while 5–27% of events occurred when no vessels were present. Threshold exceedances occurred 5–14 times more frequently when non-AIS vessels were present alone (49–78% of events) compared to the presence of only AIS vessels (5–10%). In 7–15% of above-threshold events, both AIS and non-AIS vessels were present in the study area, and it could not be determined whether one or both vessel types caused the elevated levels. Assuming that these times were dominated by noise from AIS vessels, these vessels could thereby account for up to 24% of estimated behavioural responses in porpoises (Fig. 6a). In contrast, non-AIS vessels could account for up to 85% of above-threshold events (Fig. 6c), if they were the cause of high noise when both vessel types were present. The two thresholds evaluating exceedance of noise in mid-to-high frequency noise bands (i.e. 2 and 16 kHz) were exceeded 122 and 191 times (Fig. 6b,c), while the threshold considering broadband noise was exceeded roughly 4–6 times as often (725 times; Fig. 6a). Motorised non-AIS vessels accounted for at least 49–58% of the high noise events, when thresholds were based on mid-to-high frequency bands (Fig. 6b,c). In comparison, 23% of high noise events correlated with presence of only motorised non-AIS vessels for the broadband threshold (Fig. 6a). For all three thresholds, high noise levels exceeding presumed porpoise reaction thresholds occasionally occurred when no vessels were within the study area for (5–27%), presumably due to low-frequency wind or wave noise. This interpretation is supported by the fact that this occurred more often for the broadband threshold (Fig. 6a; 27%) that includes low-frequency noise, than for the 16 kHz threshold (Fig. 6c; 5%).

## Discussion

All motorised vessels emit continuous broadband underwater noise that can substantially change the soundscape in the marine environment and negatively affect marine wildlife^1,2,23^. However, recreational vessels without AIS transmitters are currently not accounted for in the AIS-based underwater noise models used to predict the impact of vessels on underwater noise levels^13,14^. This may lead to considerable underestimations in vessel noise loads, especially in coastal areas that are important for many marine species, if recreational vessels are noisy and frequent. Here we sought to assess the potential magnitude of that underestimation by quantifying the contributions of noise from all vessels in a shallow coastal area, including both vessels with an AIS transmitter and vessels without AIS transmitters.

We show that motorised non-AIS vessels (primarily recreational vessels) contribute significant noise in the study area, with elevations in ambient noise in third-octave bands at 0.125, 2 and 16 kHz of up to 55, 47 and 51 dB, respectively (Fig. 4). Furthermore, due to their high prevalence (83% of all vessels, Fig. 1) and proximity to the coast (Figs 5, S1), recreational vessels dominated the soundscape in the three frequency bands (Fig. 4). Similar dominance of noise from recreational vessels has been reported in other studies of vessel presence in coastal areas^25,42^. We acknowledge that this ratio is highly habitat-specific, but argue that many shallow, coastal habitats with protected marine life will have comparable ratios of recreational to commercial vessels. This is largely due to the movement patterns of these vessel types, with large AIS vessels mainly travelling in offshore shipping lanes, while recreational boating occurs primarily along the coast. Our study area represents an area with dense mixed vessel traffic, close to a shipping lane (approx. 1.5 km; Supplementary Fig. S2) and >10 km from nearby marinas. This site was chosen to deliberately bias the results towards AIS vessels, highlighting that other habitats, which are further from shipping lanes and closer to marinas, likely experience an even higher ratio of non-AIS vessels to AIS vessels.

Noise received from a vessel is a complex function of its range, speed, aspect and build. However, when comparing TOLs recorded during presence of non-AIS and AIS vessels, while accounting for vessel range to the recorder, GLMMs (Fig. 5) show that noise in the 0.125, 2 and 16 kHz bands did not differ significantly between the two vessel types. The similar noise emissions at higher frequencies (2 and 16 kHz) for the two vessel types are consistent with findings of less than 10 dB difference at high frequencies (>10 kHz) for smaller vessels compared to large commercial vessels^44^. The similar TOLs at 0.125 kHz, regardless of vessel types, are likely in part due to the shallow water depth that results in a poor propagation of low-frequency sounds^23,31^ that larger vessels likely produce more of. This interpretation is supported by the fact that range was not a significant predictor for TOLs at 0.125 kHz. Furthermore, TOLs at 0.125 kHz did not consistently correlate with vessel presence (Figs 2, S7) and noise in this low-frequency band was found to be a poor predictor of noise at higher frequencies (Supplementary Fig. S8). The latter was mainly due to the shallow water depths causing poor propagation of noise at 0.125 Hz. Noise quantified in bands at lower frequencies than 0.125 kHz, i.e. with longer wavelengths such as the 0.063 kHz band, will therefore also correlate very poorly with high frequency noise. These findings support the notion that the European MSFD bands at 0.063 and 0.125 kHz^40^ are poor proxies for vessel noise loads at mid-to-high frequencies in shallow water environments, and therefore of little use in assessing acoustic habitat quality for small marine mammals in shallow waters^45^. Instead, vessel noise should be estimated at higher frequencies^45^, as also acknowledged by the European Technical Subgroup on Underwater Noise^46^, to obtain ecologically relevant measures of vessel noise impacts on species with mid-to-high frequency hearing. Our results suggest that a TOL centred at 2 kHz, as proposed by the BIAS project^9^, is a better predictor of vessel noise at higher frequencies (here 16 kHz; Supplementary Fig. S8).

Collectively, we show that current management efforts, which are focused on large commercial vessels with AIS and their low-frequency noise emissions^10,40^, may significantly underestimate actual vessel noise impacts in coastal habitats of importance to a wide range of marine species. Underestimates of vessel noise levels at higher frequencies may be especially relevant for impact assessments of small toothed whales. These species rely on sound for vital functions including navigation, foraging and communication, and they have their best hearing at mid-to-high frequencies^1,23^. Here, the harbour porpoise was chosen as a model species for evaluating the importance of small recreational vessels, when predicting potential noise impacts on marine wildlife. We used response thresholds of porpoises from three published studies^35,41,43^ to determine how often noise-induced behavioural responses might be caused by vessel passes. We find that porpoises co-located with the noise recorder would have experienced between 191 and 725 above-threshold events throughout the recording period, of which 64–95% were associated with vessel presence (Fig. 6). This suggests that in worst cases (Fig. 6a) and assuming a stationary porpoise, vessel noise in this area could have elicited a behavioural response approximately every four minutes (average 16 events/hour), unless the animal displace from the area or habituate. Results from Dyndo *et al*.^41^ suggest that porpoises may not habituate to noise from at least some vessels, despite repeated exposures. The majority of the above-threshold events occurred when only motorised non-AIS vessels were present. This suggest that the high density of motorised non-AIS vessels in shallow water, together with their typically high speeds and broadband noise emissions (Figs 1 and 3), cause them to be the most likely source of vessel noise impacts on porpoises (Fig. 6). In comparison, AIS vessels in this area had a lower presence, moved at lower speeds and were travelling further from the coast and the recorder (Figs 5, S1). Large AIS vessels further from shore will likely move faster and therefore produce more noise at higher frequencies, but may have a smaller spatial overlap with key habitats.

In our study site, the high frequency threshold at 16 kHz^35^ was mainly exceeded by motorised non-AIS vessels (58%, Fig. 6), likely because these small vessels were often travelling at high speeds (Fig. 5), resulting in high levels of broadband cavitation noise (Fig. 3). Consistent with this, the recorded TOLs in the 2 and 16 kHz third-octave bands increased significantly with speed (0.5–0.6 dB/knot, Fig. 5; see also Supplementary Table S3), which is also supported by previous studies relating higher speeds with increased propeller cavitation noise^15,47^. High vessel speeds have been associated with more pronounced reactions in toothed whales^25,27,42,48^. Oakley *et al*.^42^ found that 75% of negative reactions in harbour porpoises were caused by high-speed vessels. This is, however, not only due to higher levels of vessel noise associated with high speeds, but likely also because high speeds cause faster rise times of received noise levels. A fast rise time decreases the time available for an animal to react by displacement to minimise noise exposure. The animal may instead detect the vessel at close range and perceive it as an immediate threat and exhibit an erratic response^27,49^. The rise time of vessel noise may be a particular issue in shallow water, where low frequency sounds, that otherwise could serve as a timely warning of an approaching noise source^48^, propagate poorly.

Besides the substantial spatial overlap between recreational vessels and noise-sensitive coastal species, the temporal patterns of these vessels may further add to the potential for significant noise impacts. Recreational boating in warmer summer months largely overlap with the breeding/spawning periods of marine species, such as harbour porpoises, which possibly make the animals more sensitive to disturbance^3^. Furthermore, the often lower ambient noise levels during warmer months (due to lower wind speeds and lower precipitation in temperate areas) will cause the noise contributions and perceived loudness from vessels to be more significant. Ultimately, the spatiotemporal overlap of recreational boating with coastal key habitats for marine species^18,20–22,34^ and their often erratic movements and high speeds, emphasise that these vessel types could have significant impacts on noise-sensitive species.

## Conclusion

Management of vessel noise first of all requires an understanding of the contributions from all relevant noise sources. Here we have shown that recreational vessels without AIS transmitters were the most frequent vessels and the dominant contributors of underwater noise in a shallow, coastal area. In Europe, 46% of coastal waters have water depths shallower than 20 m^28,29^ and may thereby similarly be dominated by mid-to-high frequency noise from the more than 6 million small, recreational vessels^12^. While acoustic modelling is undoubtedly a valuable tool for predicting vessel noise, in particular in offshore deep-water areas, we conclude that modelling on the basis of AIS data alone may lead to severe underestimations of the actual vessel noise levels and noise impacts in shallow coastal waters with dense recreational boating. When operated at high speeds, small recreational vessels produce broadband cavitation noise, which has the potential to affect a wide range of marine species for which shallow coastal areas are key habitats. We therefore propose that recreational vessels capable of planing, or with engine sizes above some set value, are equipped with an AIS-transmitter or a similar monitoring system. This will allow for inclusion of the noisiest small recreational vessels in predictive noise models, as a first step to reach better estimates of actual vessel noise loads and vessel noise impacts. Furthermore, to obtain relevant measures of noise pollution in shallow water habitats and its effects on marine species with mid-to-high frequency hearing, we encourage the implementation of a monitoring band at 2 kHz, as suggested by the BIAS group^9^, in addition to the low frequency bands stipulated in the MSFD. Finally, if adverse effects of noise are to be reduced in marine protected areas and other key habitats, imposing vessel speed limits that avoid cavitation would be an effective mitigation tool.

## Supplementary information

Supplementary Material

## Data Availability

Data available at Zenodo digital repository (www.zenodo.org), 10.5281/zenodo.3465461.
