# Supplementary material for: Recreational vessels without Automatic Identification System (AIS) dominate anthropogenic noise contributions to a shallow water soundscape
Source: Sci Rep. 2019 Oct 29;9:15477. doi: 10.1038/s41598-019-51222-9 (PMC6820791; doi:10.1038/s41598-019-51222-9)
Supplement: Supplementary file 1 — Supplementary Material [file 41598_2019_51222_MOESM1_ESM.pdf]

Electronic Supplementary Material for:

Recreational vessels without Automatic Identification System (AIS) dominate anthropogenic noise contributions to a shallow water soundscape

Line Hermannsen[1,2*], Lonnie Mikkelsen[2], Jakob Tougaard[2], Kristian Beedholm[1], Mark Johnson[3,1] & Peter T. Madsen[1,4]

[1]Zoophysiology and [2]Marine Mammal Research,

Department of Bioscience, Aarhus University, Denmark

[3]Sea Mammal Research Unit, University of St. Andrews, St. Andrews, United Kingdom

[4]Aarhus Institute of Advanced Studies, Aarhus University, Aarhus, Denmark.

[*]Corresponding author. Email: lihe@bios.au.dk

Supplementary methods

Study area

The presence of porpoises was based on knowledge from Mikkelsen et al. (2017), but also confirmed by visual observations of animals during fieldwork and detection of porpoise clicks in sound recordings. The study area was approx. 20 km from the city Aarhus with the largest commercial harbour in Denmark, and 10 km from Ebeltoft ferry harbour. Nine marinas, incl. two in Aarhus, are located between 13 and 20 km from the study area. The main shipping route connecting Aarhus to Kattegat runs approx. 1.5 km south of the recording station.

Recorder deployment

The recorder was suspended 1.5 m from the seabed between an anchor and a mid-water buoy with the hydrophone facing upwards. To maintain visibility of the recorder position from the tracking point, but minimize artificial noise and drag of the recording gear, a surface buoy with a slack 10 m rope was attached to the mid-water buoy.

Theodolite tracking

The theodolite was placed at the same location every day at the high point *Burs klint* (56°6.153N, 10°32.23E, 47 m above sea level), where there was a good overview of the marine study area. To ensure that the exact position of the theodolite was known, the position of a lighthouse (Hjelm Lighthouse, 56°8.00N 10°48.29E) and a reference pole (measured by differential GPS by a certified surveyor in 2015, as used in Mikkelsen et al. 2017) were used daily as fix points. A laptop running the software Cyclops Tracker (version 2004, Eric Kniest, University of Newcastle, Australia) delivered real-time display of the tracked vessels via a communication port connection to the theodolite, and stored the data for later processing. Variations in sea level according to tide were corrected for in Cyclops Tracker by tracking sea level on a tide pole approx. every hour. In case of multiple vessels present at the same time, tracking of the motorised vessel closest to the recorder was prioritised. The 2 km criteria led to discard of 26% of the theodolite tracked vessels.

Vessel types

Registered vessels within the study area (max 2 km from the recorder) and the study period were grouped into nine types (Table S1): Cargo vessels, Tankers, Dredgers, Other vessels (e.g. fishing vessels and navy vessels), Motorboats, Speedboats, Rigid-hulled inflatable boats (RHIBs), Dinghies, Sailboats. Vessels tracked by AIS were automatically labelled with vessel type in the AIS data, whereas theodolite tracked vessels without AIS were instead labelled manually during observation and confirmed later from photos. Here, motorboats were characterised by having a cabin, whereas speedboats were not fitted

with a cabin, but had a windshield in the front. Dinghies were characterised as open aluminium boats with small outboard engines and without cabin or windshield. All inflatable boats were grouped in the category 'RHIBs'. Sailboats were all vessels that had sails, including those that were travelling by motor power (including a catamaran and a wooden ship with masts).

Vessel tracks

Multiple passes of the same vessel were treated as separate events, as there were sufficient distinct vessels to dilute any resulting pseudo replication. AIS vessel tracks always extended beyond the 2 km criterion, as we had collected available AIS data for a 20 km area around our recorder. We did not extrapolate tracks of non-AIS vessels out to 2 km, as recreational vessels often travel unpredictably (Nowacek et al., 2001) and due to acoustic shading by the sandbank of many of them. Vessel tracks were also used to overlay AIS data and theodolite data in time and space to determine if a small vessel had voluntarily installed an AIS (Supplementary Fig. S4). If both were available for a given vessel, the AIS data was used for further analysis. The comparison between the theodolite and AIS track for unique vessels allowed for estimating the difference between the two measures, and was used to obtain an error estimate for theodolite positions (assuming that AIS positions were correct).

Harbour porpoise reaction thresholds

We used response thresholds of porpoises derived from three previous studies reporting avoidance, porpoising and decreased foraging (Dyndo et al. 2015, Tougaard et al. 2015 and Wisniewska et al. 2018), to find high noise events above these response thresholds and correlate with vessels present at those times. The first threshold was taken from a study by Dyndo et al. (2015) which documented porpoises reacting by increased porpoising behaviour to M-weighted (Southall et al. 2007) broadband (25 Hz – 80 kHz) vessel noise at levels > 123 dB re 1µPa (RMS, 30 s window). To use this threshold, we applied the M-weighting to the TOLs and calculated the RMS received level over 30 s bins summing the power across the 36 third-octave bands from 25 Hz to 80 kHz. The second threshold was taken from Tougaard et al. (2015), who gathered data from several studies of porpoise reactions to short impulse noise (quantified as RMSfast, over 125 ms) at different frequencies. They found that reaction thresholds of negative phonotaxis fit well with the curve shape of a porpoise audiogram (Kastelein et al. 2010), although shifted 45 dB above the audiogram. We calculate the thresholds by adding 45 dB to the porpoise hearing thresholds for 300 ms pure-tones (Kastelein et al. 2010). Events were considered to potentially elicit behavioural responses in porpoises, if noise in the 2 or 16 kHz third-octave band exceeded the hearing threshold by ≥ 45 dB. Although a 1 second averaging time was used instead of the 125 ms (RMSfast) in Tougaard et al. (2015), these will be little different given the relatively slow changes in vessel noise over a second. The third threshold was taken from Wisniewska et al. (2018) who investigated how tagged wild porpoises reacted to vessel noise. These authors found a significant

reduction in foraging when noise levels exceeded 96 dB re 1µPa (RMS) in a third-octave band at 16 kHz, quantified as the 90th percentile of noise in 0.5 s bins over 1 min windows. To implement this threshold, we calculated the 90th percentile of 1 s bins over a 1 min sliding window. For all thresholds, a blanking time was applied such that, if a high noise level was detected, a subsequent detection could not be made until the noise level had been below the threshold for at least 1 minute. This blanking time was used to avoid that a vessel pass counted multiple times, as it was expected that an animal would react during vessel approach phases, when noise levels initially increased and exceeded porpoise reaction thresholds.

An index of vessel noise (Jakob Tougaard 2016)

The received noise level (RL) from a single vessel at range r1 can be modelled as:

$$RL_1 = SL_1 - \kappa \log_{10} r_1 \qquad (1)$$

Where SL is the source level and absorption is ignored.

If there are N vessels, at respective ranges r_1, r_2,… r_N, the combined received level, expressed as intensity, is given as:

$$RL = 10 \log_{10}\left(10^{\frac{RL_1}{10}} + 10^{\frac{RL_2}{10}} + \cdots 10^{\frac{RL_N}{10}}\right) \qquad (2)$$

Combining with (1) yields:

$$RL = 10 \log_{10}\left(10^{\frac{(SL_1 - \kappa \log_{10} r_1)}{10}} + 10^{\frac{(SL_2 - \kappa \log_{10} r_2)}{10}} + \cdots 10^{\frac{(SL_N - \kappa \log_{10} r_N)}{10}}\right) \quad (3)$$

For simplicity, we assume that all vessels have the same SL and rearrange:

$$RL = 10 \log_{10}\left(10^{\left(\frac{SL}{10} - \frac{\kappa \log_{10} r_1}{10}\right)} + 10^{\left(\frac{SL}{10} - \frac{\kappa \log_{10} r_2}{10}\right)} + \cdots 10^{\left(\frac{SL}{10} - \frac{\kappa \log_{10} r_N}{10}\right)}\right) =$$

$$10 \log_{10}\left(\frac{10^{\frac{SL}{10}}}{10^{\frac{\kappa \log_{10} r_1}{10}}} + \frac{10^{\frac{SL}{10}}}{10^{\frac{\kappa \log_{10} r_2}{10}}} + \cdots \frac{10^{\frac{SL}{10}}}{10^{\frac{\kappa \log_{10} r_N}{10}}}\right) =$$

$$10 \log_{10}\left(10^{\frac{SL}{10}}\left(\frac{1}{10^{\frac{\kappa \log_{10} r_1}{10}}} + \frac{1}{10^{\frac{\kappa \log_{10} r_2}{10}}} + \cdots \frac{1}{10^{\frac{\kappa \log_{10} r_N}{10}}}\right)\right)$$

As $10 \log_{10} 10^{\frac{SL}{10}} = SL$ and $10^{\frac{\kappa \log_{10} r}{10}} = \left(10^{\log_{10} r}\right)^{\kappa/10} = r^{\kappa/10}$ we obtain:

$$RL = SL + 10 \log_{10}\left(\frac{1}{r_1^{\kappa/10}} + \frac{1}{r_2^{\kappa/10}} + \cdots \frac{1}{r_N^{\kappa/10}}\right) = SL + 10 \log_{10} \sum \frac{1}{r_i^{\kappa/10}} \qquad (4)$$

The figure below shows a simple example, where one vessel is stationary at 1000 m and another varies from 100 m to 10 km. A simple assumption that the RL is determined by the closest vessel will at most be 3 dB off, when the two vessels are at the same range from the receiver.

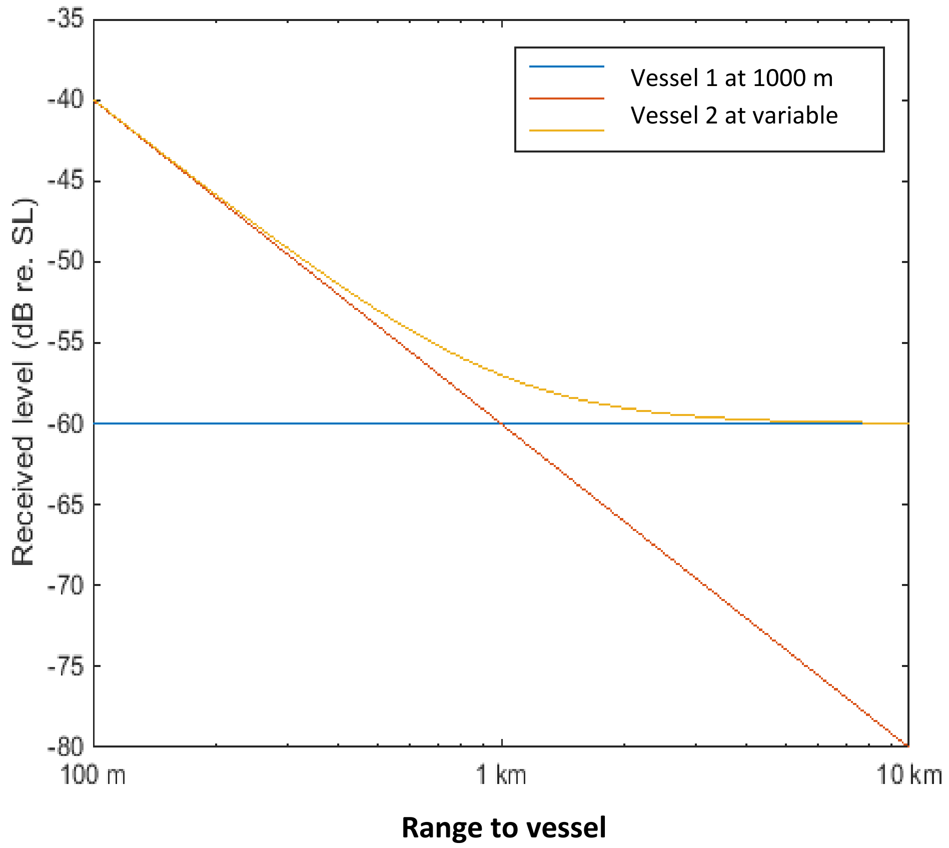

Supplementary Figures and Tables

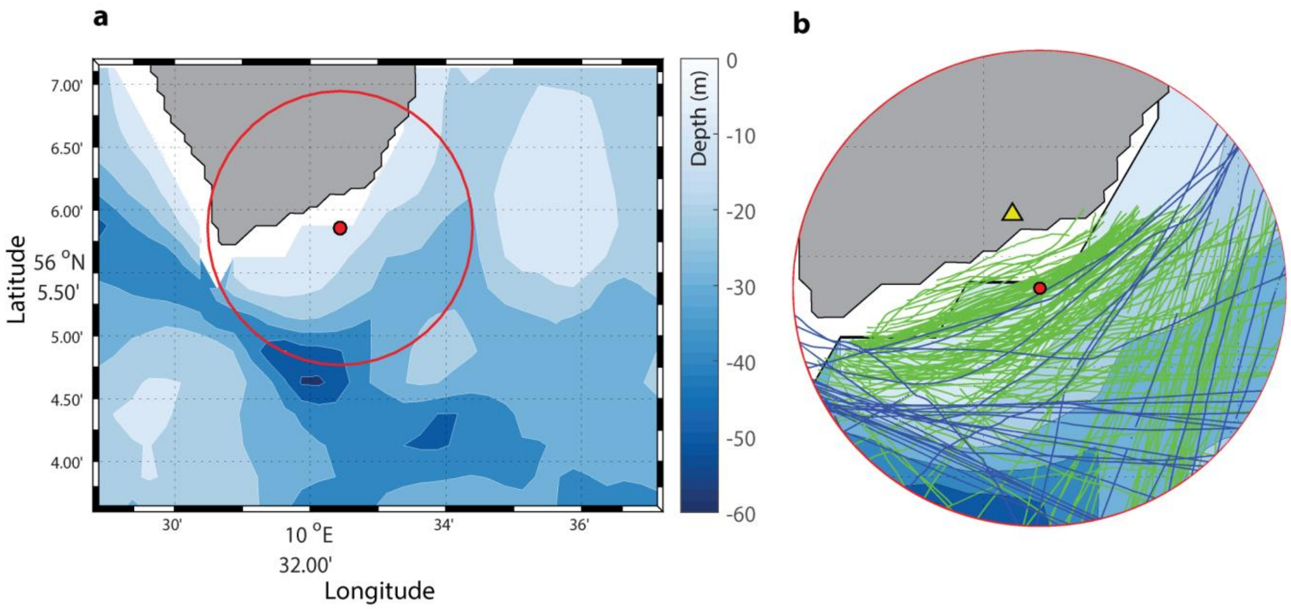

Figure S1. Water depth (a) and vessel passes (b) within the study area, i.e. red circle = 2 km around sound recorder (red filled circle). The yellow triangle on (b) marks the position of the theodolite, while green lines mark interpolated tracks of non-AIS vessels and blue lines mark AIS vessel tracks.

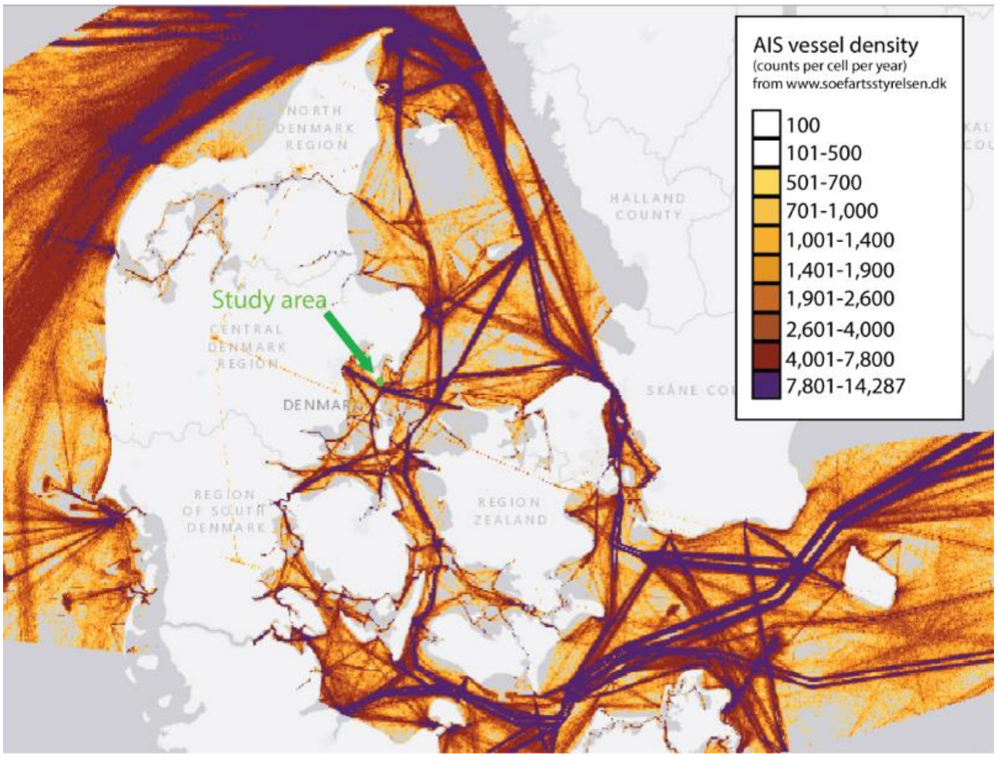

Figure S2. Map of AIS vessel density in Danish waters during 2016. Map from the Danish Maritime Authority (www.soefartsstyrelsen.dk). The position of the study area is shown in green.

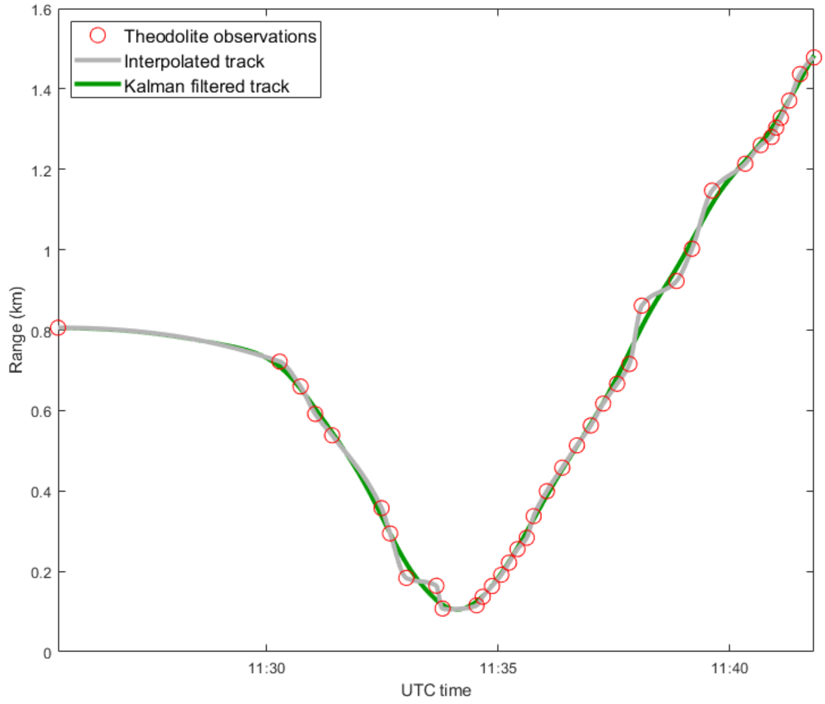

Figure S3. Example of interpolation and kalman filtration of theodolite observations of a vessel.

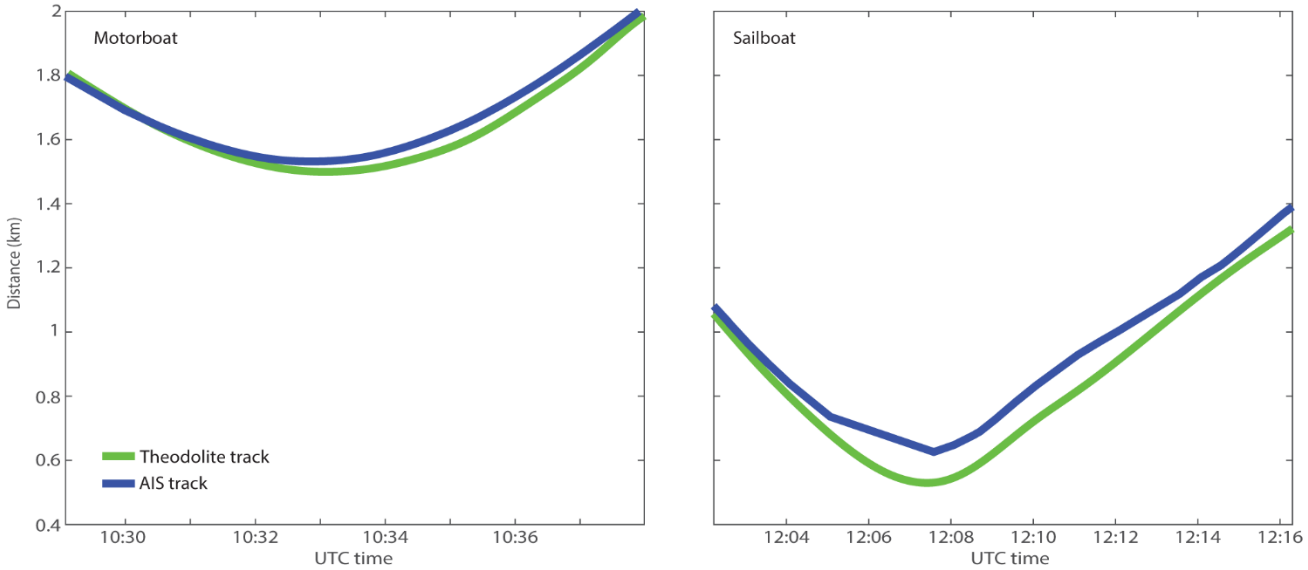

Figure S4. Examples of recreational vessels that were tracked with theodolite, but were also found to have an AIS by overlaying theodolite tracks with AIS tracks.

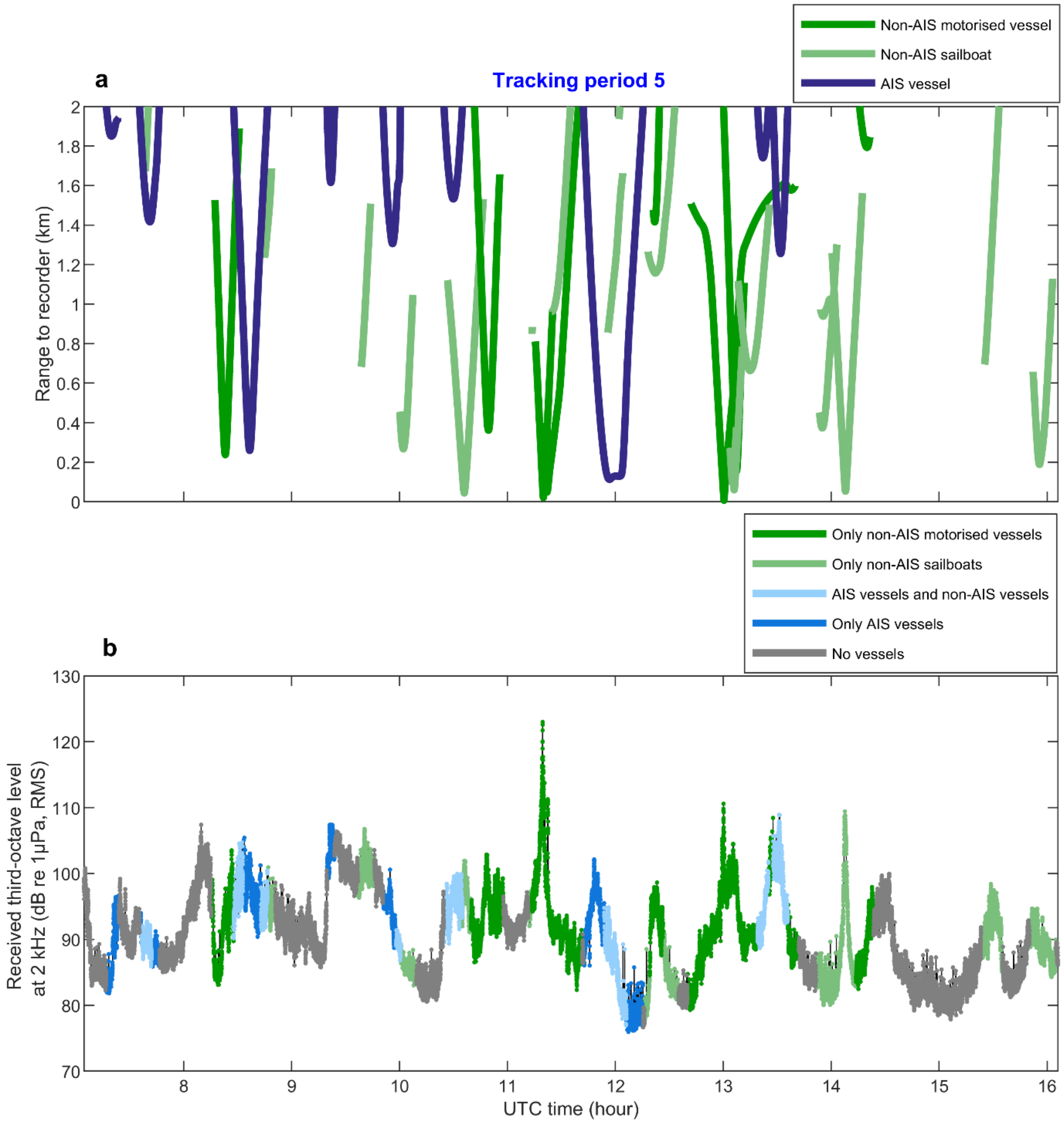

Figure S5. Examples of how recorded TOLs (bottom, centred at 2 kHz) were divided into the five vessel categories (b), depending on vessel presence in the study area (a).

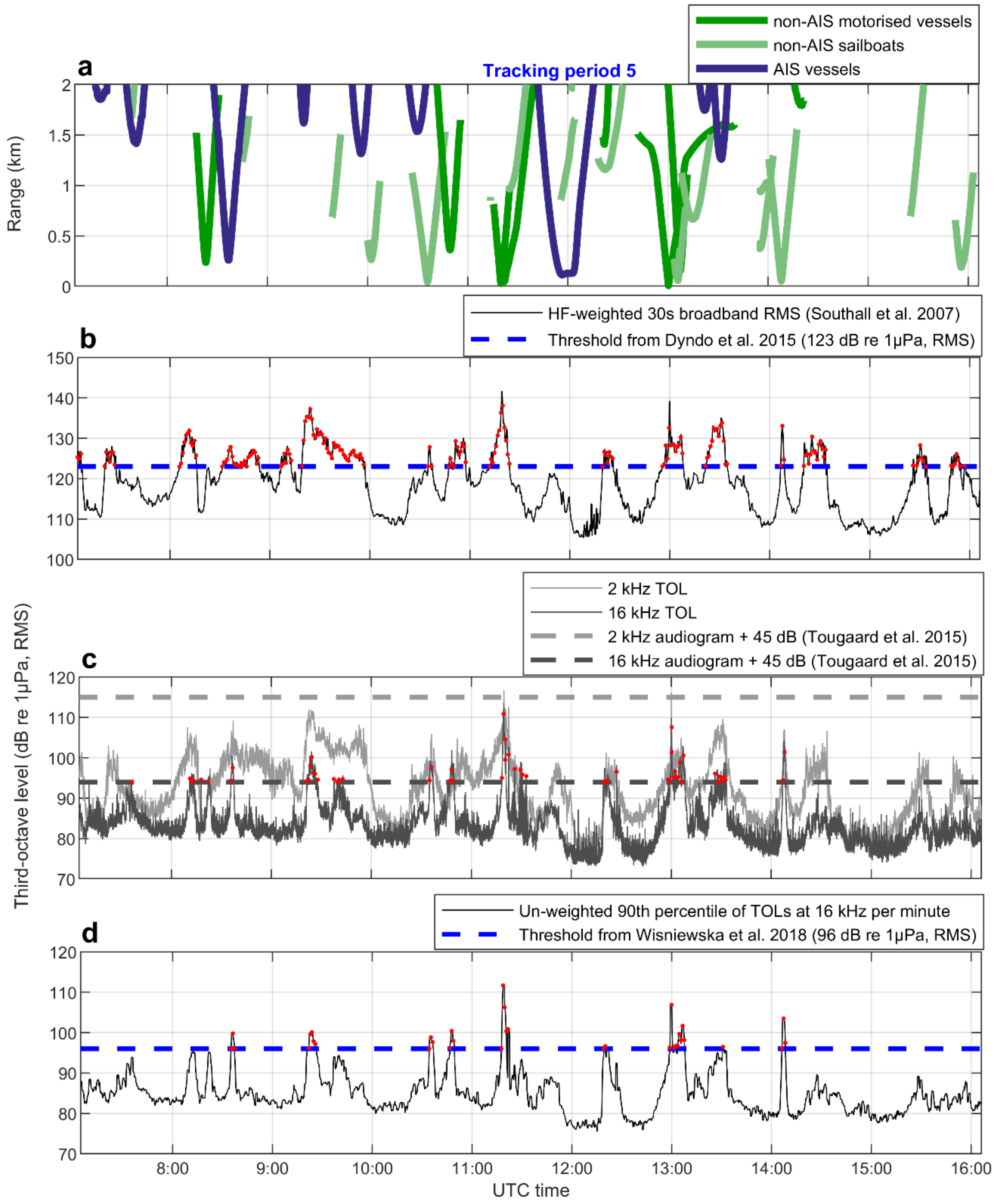

Figure S6. Examples of how exceedance of the three response thresholds for porpoises (b-d) in Fig. 6 were found (red dots), using a blanking time of 1 minute (i.e. max one exceedance pr. minute).

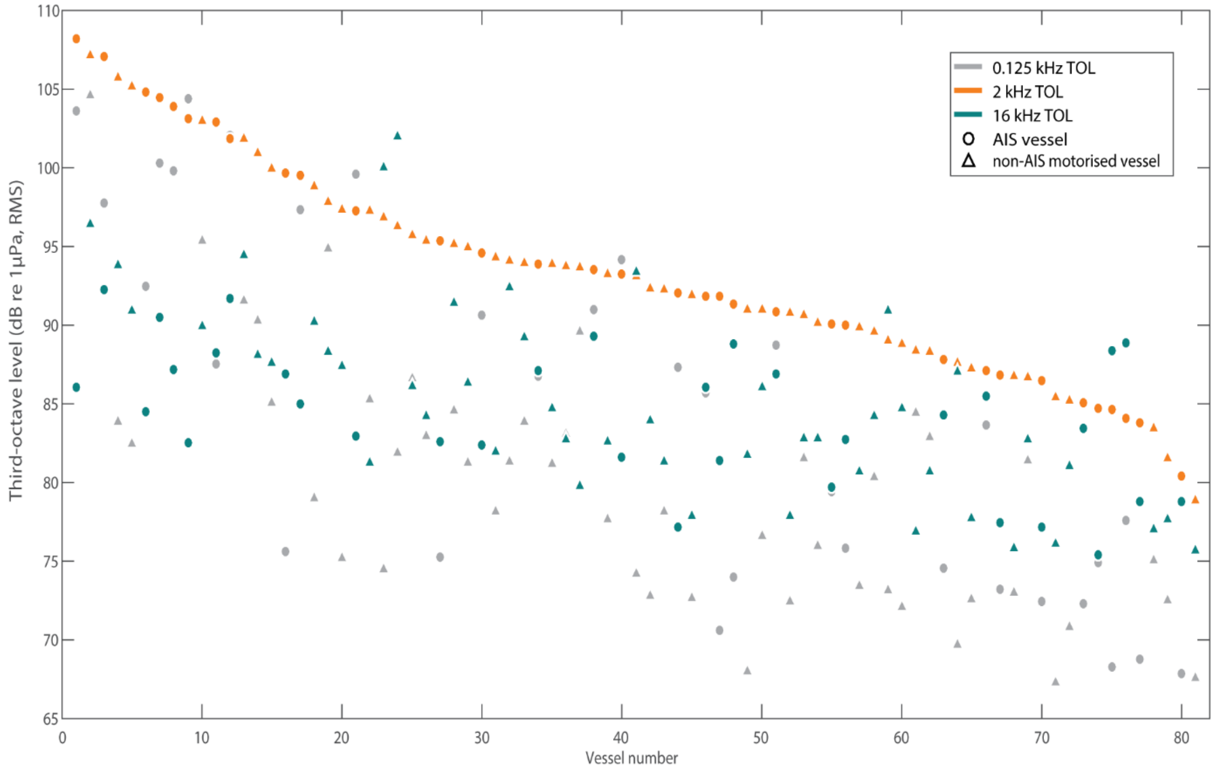

Figure S7. TOLs at 0.125 (grey), 2 (orange) and 16 kHz (green) during the closest point of approach (CPA) for each vessel. Vessel types are AIS vessels (circle) and non-AIS motorised vessels (triangle). Data is sorted by the levels in the 2 kHz band on the x-axis (from high to low).

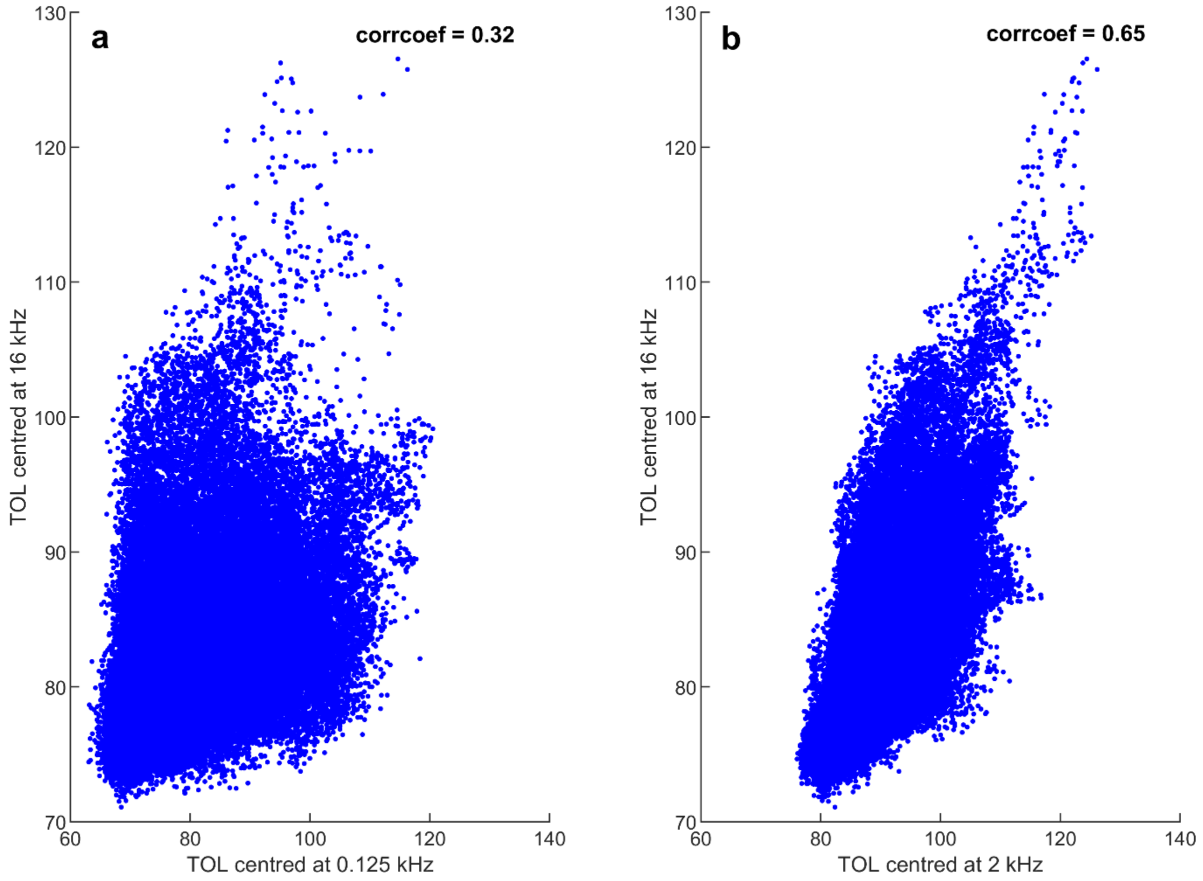

Figure S8. The correlation of 16 kHz TOLs with TOLs at 0.125 kHz (a) and 2 kHz (b). Data points are for the full study period. Pearson's correlation coefficients are shown in top of the plots.

Table S1. Division of time according to vessel presence within 2 km of the underwater noise recorder.
[1]At least one non-AIS vessel was present together with AIS vessels during these time periods.
[2]These vessel groups could be present in the given time period category, however the grouping was based on either AIS vessels (category 2) or motorised non-AIS vessels (category 3) being present in the study area.

	**Time period category**	**Hours**	**AIS vessels present?**	**Non-AIS motorised vessels present?**	**Non-AIS sailboats present?** (engine on/off)
**1**	Only AIS vessels	2.08	Yes	No	No
**2**	AIS vessels and non-AIS vessels[1]	4.45	Yes	Maybe[2]	Maybe[2]
**3**	Only non-AIS vessels motorised (without masts)	8.14	No	Yes	Maybe[2]
**4**	Only non-AIS sailboats (with masts)	13.90	No	No	Yes
**5**	No vessels	6.75	No	No	No

Table S2. The number of AIS and non-AIS vessels in the study area divided into 9 vessel types (see vessel type definitions in Supplementary methods). Some small vessels with no AIS requirement had voluntarily installed an AIS on board.

	**Vessel type**	**Total**	**Vessels with AIS**	**Vessels without AIS (non-AIS)**
**AIS required**	Cargo/tanker/dredger	11/2/2	11/2/2	0
**AIS not required**	Motorboat	35	2	33
	Speedboat/RHIB/dinghy	9/2/2	0	9/2/2
	Other vessel	4	2	2
	Sailboat	131	15	116
	AIS required	15	15	0
	AIS not required	183	19	164
	**Total**	**198**	**34**	**164**

Table S3. Results for generalized linear mixed-effects models (GLMMs) using data from Figure 5 for third-octave levels (TOLs) centred at 0.125, 2 and 16 kHz. Model specifications: *Noise ~ 1 + Range*Type + Type*Speed + (1|Day)*. Degrees of freedom = 53.

		**Estimate**	**SE**	**tStat**	**pValue**	**Lower**	**Upper**
**0.125 kHz**	Intercept	95.85	7.74	12.381	2.87e-17*	80.32	111.37
	Range	-0.85	6.16	-0.138	0.891	-13.20	11.50
	Type_2 (non-AIS)	-0.06	8.36	-0.007	0.994	-16.83	16.70
	Speed	0.56	0.32	1.778	0.081	-0.07	1.20
	Range:Type_2	-6.83	6.59	-1.036	0.305	-20.06	6.40
	Type_2:Speed	-0.54	0.34	-1.574	0.121	-1.23	0.15
**2 kHz**	Intercept	84.90	5.02	16.918	5.11e-17*	74.84	94.97
	Range	-15.85	3.48	-4.56	**3.08e-05***	-22.83	-8.88
	Type_2 (non-AIS)	3.86	4.55	0.85	0.400	-5.26	12.99
	Speed	0.56	0.17	3.24	**0.002***	0.21	0.90
	Range:Type_2	5.12	3.76	1.36	0.179	-2.41	12.66
	Type_2:Speed	-0.22	0.19	-1.17	0.246	-0.60	0.16
**16 kHz**	Intercept	75.86	6.52	11.64	3.22e-16	62.78	88.93
	Range	-16.21	4.67	-3.47	**0.001***	-25.58	-6.83
	Type_2 (non-AIS)	1.48	6.12	0.24	0.809	-10.79	13.76
	Speed	0.48	0.23	2.09	**0.041***	0.02	0.95
	Range:Type_2	3.86	5.05	0.76	0.448	-6.27	13.98
	Type_2:Speed	-0.03	0.25	-0.12	0.901	-0.54	0.48
